# Loss of *p19Arf* promotes fibroblast survival during leucine deprivation

**DOI:** 10.1242/bio.058728

**Published:** 2022-02-17

**Authors:** Kerry C. Roby, Allyson Lieberman, Bang-Jin Kim, Nicole Zaragoza Rodríguez, Jessica M. Posimo, Tiffany Tsang, Ioannis I. Verginadis, Ellen Puré, Donita C. Brady, Constantinos Koumenis, Sandra Ryeom

**Affiliations:** 1 Department of Cancer Biology, University of Pennsylvania, Philadelphia, PA 19104, USA; 2 Cell and Molecular Biology Graduate Group, University of Pennsylvania, Philadelphia, PA 19104, USA; 3 Department of Radiation Oncology, Perelman School of Medicine, University of Pennsylvania, Philadelphia, PA 19104, USA; 4 Department of Biomedical Sciences, University of Pennsylvania, School of Veterinary Medicine, Philadelphia, PA 19104, USA; 5 Abramson Family Cancer Research Institute, Perelman School of Medicine, University of Pennsylvania, Philadelphia, PA 19104, USA

**Keywords:** *P19Arf*, Fibroblast, Leucine deprivation, Integrated stress response, Autophagy

## Abstract

Fibroblasts are quiescent and tumor suppressive in nature but become activated in wound healing and cancer. The response of fibroblasts to cellular stress has not been extensively investigated, however the *p53* tumor suppressor has been shown to be activated in fibroblasts during nutrient deprivation. Since the p19 Alternative reading frame (*p19Arf*) tumor suppressor is a key regulator of p53 activation during oncogenic stress, we investigated the role of p19^Arf^ in fibroblasts during nutrient deprivation. Here, we show that prolonged leucine deprivation results in increased expression and nuclear localization of *p19Arf*, triggering apoptosis in primary murine adult lung fibroblasts (ALFs). In contrast, the absence of *p19Arf* during long-term leucine deprivation resulted in increased ALF proliferation, migration and survival through upregulation of the Integrated Stress Response pathway and increased autophagic flux. Our data implicates a new role for *p19Arf* in response to nutrient deprivation.

This article has an associated First Person interview with the first author of the paper.

## Abstract (precis)

**Summary:** We investigated the response of adult lung fibroblasts to nutrient deprivation and reveal a role for the *p19Arf* tumor suppressor in suppressing both fibroblast activity and survival during leucine deprivation.

## INTRODUCTION

The tumor microenvironment (TME) is a mixed population of cells that includes fibroblasts, endothelial cells, and immune cells surrounding the tumor (Hanahan and Weinberg, 2011). Although originally thought to be genetically stable, cells in the TME are not ‘normal’ and promote tumorigenesis (Fiori et al., 2019). Fibroblasts are typically a quiescent cell type that becomes activated in response to a range of stimuli including stress and injury (Kalluri, 2016). During wound healing, fibroblasts are responsible for releasing matrix metalloproteases to clear damage from the wound and deposit matrix and collagen to promote healing. Activated fibroblasts in the TME also produce extracellular matrix that surrounds the tumor and limits the efficacy of cancer therapies due to generation of a desmoplastic stroma and reduced access to tumor cells (Julia et al., 2013). As a result of the hyperproliferative nature of cancer cells, cells in the TME are also exposed to stressors including acidosis, hypoxia, nutrient deprivation, and amino acid deprivation (AAD) (Hanahan and Weinberg, 2011), activating the integrated stress response (ISR) pathway. AAD results in the accumulation of uncharged tRNAs that bind the histidyl-tRNA synthetase-related domain of the general control nonderepressible 2 (GCN2) kinase of the ISR pathway (Lehman et al., 2015; Tang et al., 2015; Battu et al., 2017). Activated GCN2 phosphorylates the eukaryotic translation initiation factor-2 alpha (eIF2α), which inhibits global protein translation, except for factors that allow cells to cope with the stress, such as the pro-apoptotic genes PUMA and CHOP (Pakos-Zebrucka, et al., 2016).

Tumor cells subjected to stress activate the ISR to induce pro-survival programs including autophagy and senescence, rather than apoptosis (B'Chir et al., 2013; Mejlvang et al., 2018). However, autophagy is viewed as a double-edged sword, with studies suggesting both anti- and pro-tumorigenic effects (White and DiPaola, 2009; Asha and Sharma-Walia, 2018; Gwangwa et al., 2018). Activation of the GCN2/ATF4 pathway in cancer cells promotes tumorigenesis, while targeted deletion of ATF4 and/or GCN2 resulted in apoptosis and tumor regression (Ye et al., 2010). In contrast to tumor cells, normal cells undergo cell cycle arrest in response to stress by upregulation of the *p53* target gene, *p21* (Karimian et al., 2016). It has been previously shown that *p21* mRNA levels are upregulated in cancer cells in response to AAD, including leucine deprivation in a p53-dependent manner (Tang et al., 2015). TP53 activation is regulated by the MDM2 ubiquitin ligase and in response to oncogenic stress, the *p19Arf* tumor suppressor sequesters MDM2, thereby preventing it from targeting p53 for degradation permitting the transactivation of p53-dependent genes to inhibit cell cycle progression among other functions (Stott et al., 1998; Weber et al., 2000; Sherr, 2006). *P19Arf* is the mouse homolog of human *P14ARF* and functions as a tumor suppressor in a p53-dependent manner to induce apoptosis, cell cycle arrest, and autophagy (Suzuki et al., 2003; Itahana and Zhang, 2008; Sharpless and Sherr, 2015). A limited number of studies have investigated the role of *p19Arf* in the context of the ISR pathway, with one study showing that deletion of the ISR-regulated gene *ATF4* increased the expression of the *CDKN2A* genes *p16Ink4a* and *p19Arf* suppressing colony formation and tumor growth (Horiguchi et al., 2012).

Fibroblasts in the tumor microenvironment are referred to as cancer associated fibroblasts (CAFs) and are activated by tumor cells and endothelial cells promoting tumorigenesis by increased deposition and remodeling of ECM that favors the metastatic outgrowth of tumor cells (Beacham and Cukierman, 2005), (Wang et al., 2017). CAFs express fibroblast activation protein (FAP) with studies showing that deletion of FAP resulted in increased collagen deposition and reduced tumor growth in a mouse model of lung cancer (Santos et al., 2009). As residents of the TME, they are also subjected to stress present in the tumor microenvironment and use different mechanisms to adapt. For example, CAFs have been shown to use autophagy to promote cancer cell growth and tumor progression in head and neck squamous cell carcinoma (New et al., 2018). However, CAFs have also been shown to enhance tumor progression through metabolic reprogramming by the ISR. In autophagy, ubiquitinated proteins and organelles are shuttled to the developing autophagolysosome to be degraded by autophagy cargo receptors including p62, whose deletion has been shown to enhance tumor progression (Linares et al., 2017). Although the tumor suppressive functions of *p19Arf* have been well characterized, its role in CAFs and in response to stress and the ISR pathway have not yet been investigated. While genetic mutations of CAFs are rare (Qiu et al., 2008), epigenetic changes in CAFs in breast cancer have been shown to increase fibroblast function and tumor growth rates when co-injected in xenograft models that have genetically stable p53 (Arandkar et al., 2018).

In this study, we investigated the role of *p19Arf* in fibroblasts in response to nutrient deprivation. We show increased tumor growth of transplanted syngeneic sarcoma and lung cancer cells in *p19Arf^−/−^* mice with *p19Arf* deleted in host cells including fibroblasts. Our data indicate that long-term AAD induces p19^Arf^ expression in primary murine adult lung fibroblasts (ALFs). However, in the absence of *p19Arf,* ALFs show increased proliferation, activation, and survival during prolonged AAD due in part to increased autophagic flux.

## RESULTS

### P19^Arf^ is induced in fibroblasts in response to amino acid deprivation

**Fig. 1.**
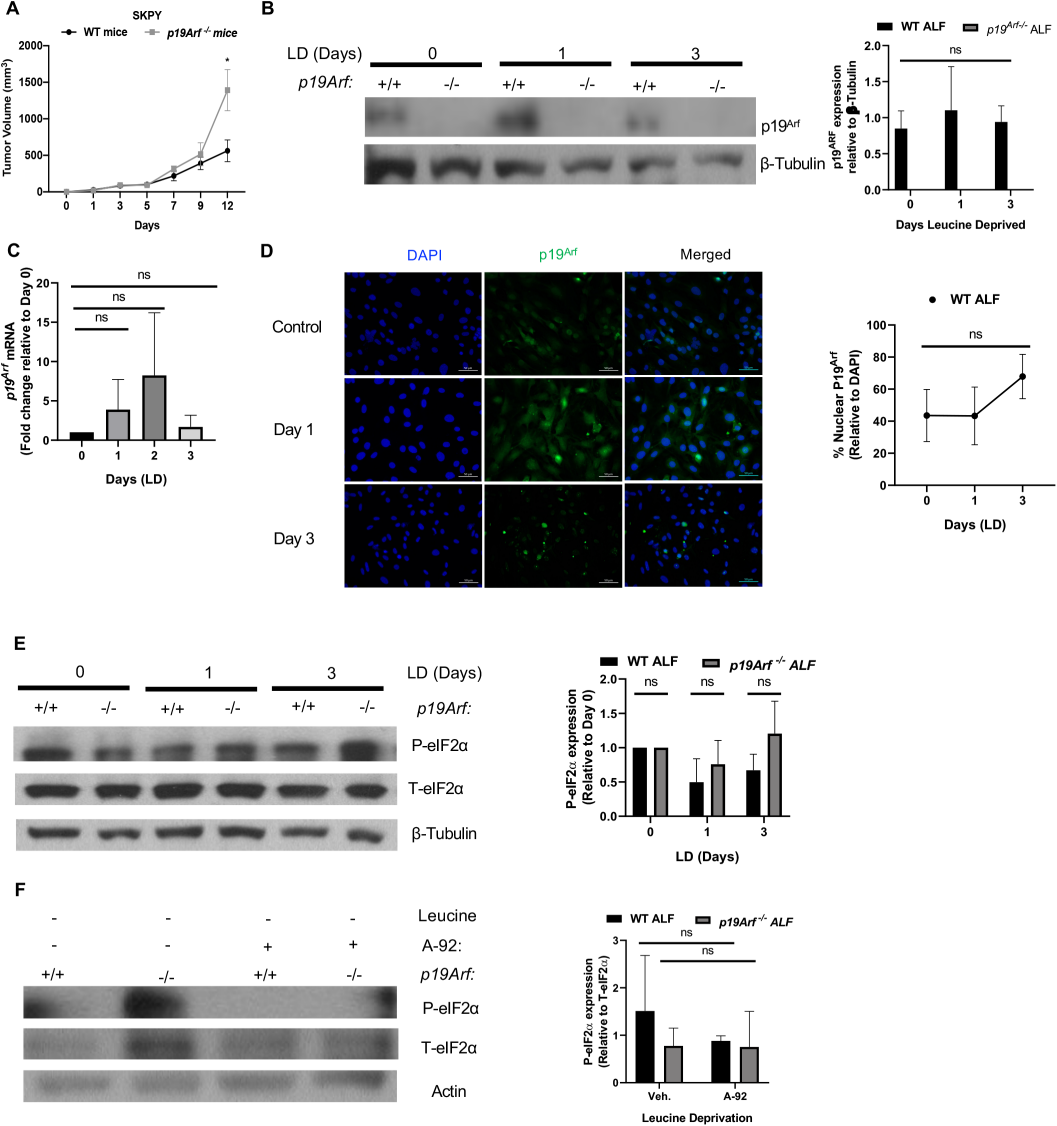
**Loss of *P19Arf* enhances tumor growth and primary lung fibroblasts induce p19^Arf^ in response to leucine deprivation (LD).** (A) Graph of sarcoma (SKPY) tumor volume on the indicated days. SKPY cells were injected into the flank of WT and *p19Arf-null* mice. Tumor volume was measured by caliper. *N*=7 mice/WT cohort and *N*=15 mice/*p19Arf^−/−^* cohort. (B) Western blot for p19^Arf^ protein expression in WT and *Arf-null* fibroblasts after LD for the indicated days. Densitometric quantification of p19^Arf^ protein expression relative to β-tubulin is shown on right. (C) qPCR for *p19Arf* mRNA in WT or *p19Arf^−/−^* fibroblasts during LD for the indicated days. (D) Representative immunofluorescence images for P19^Arf^ subcellular localization on the indicated days after LD in WT or *p19Arf^−/−^* ALFs. Quantification of nuclear p19^Arf^ is shown in graph on right. (E) Western blot analysis of phospho-eIF2α (P-eIF2α) and total eIF2α expression in WT and *p19Arf*-null murine ALFs during LD for the indicated days. Graph quantifies intensity of P-eIF2α expression relative to day 0. β-Tubulin used as loading control. (F) Western blot analysis of P-eIF2α expression in WT and *p19Arf^−/−^* ALFs upon treatment with a GCN2 inhibitor (GCN2-IN-1; A-92 1 µM in DMSO) during overnight LD. Actin used as a loading control. Quantification of P-eIF2α expression relative to total eIF2α. *N*=3; *, *P*<0.05; ns, not significant.

The tumor suppressor P19^Arf^ has been well characterized and shown to be downregulated or silenced across different tumor types (Ishii et al., 1999; Ozenne et al., 2010). Oncogenic stress and DNA damage are known to induce P19^Arf^ in tumor cells, which in turn stabilizes and activates p53 (Zindy et al., 2003; Cleveland and Sherr, 2004; Sherr, 2006), but the role of these tumor suppressors in other cellular populations in the tumor microenvironment has not been extensively examined. Syngeneic sarcoma cells driven by oncogenic Kras (SKPY) were inoculated in the flanks of wild-type (WT) or *p19Arf^−/−^* mice with *p19Arf* deleted in host cells (Fig. 1A). Tumor growth was increased in *p19Arf^−/−^* mice, implicating a tumor suppressive role for p19^Arf^ in cells in the tumor microenvironment. We focused on fibroblasts and subjected WT and *p19Arf-*null ALFs to leucine deprivation (Sheen et al., 2011; Xiao et al., 2016), revealing a trend of increased *p19Arf* expression at the protein (Fig. 1B) and mRNA levels (Fig. 1C) after 24 h that was sustained long term over 2-3 days. In addition, leucine deprivation also resulted in a trend of increased nuclear localization of p19^Arf^ in ALFs (Fig. 1D), where it induces its cellular functions.

To cope with AAD, cells activate the ISR exclusively through the GCN2 arm of the pathway, resulting in phosphorylation of eIF2α and nuclear localization of ATF4 (Pakos-Zebrucka et al., 2016). We probed for activation of the ISR in WT and *p19Arf^−/−^* ALFs and show a trend of increased phospho-eIF2α (P-eIF2α) in *p19Arf*^−/−^ fibroblasts at baseline and after prolonged leucine deprivation indicating ISR pathway activation (Fig. 1E). Fibroblasts grown on stiff tissue culture plastic are activated in the absence of stimuli thus some level of ISR activation before leucine deprivation is not surprising (Skardal et al., 2013). To confirm dependence on GCN2 activity in response to AAD, we treated fibroblasts with a GCN2 inhibitor (A-92) (Ying and Khaperskyy, 2020) and probed for P-eIF2α by western blot in response to leucine deprivation (Fig. 1F). We show that phosphorylation of eIF2α in both WT and *p19Arf-*null fibroblasts is lost when cells are treated with a GCN2 inhibitor during leucine deprivation, confirming increased GCN2 activation in response to amino acid deprivation in *p19Arf^−/−^* ALFs (Fig. 1E). These data suggest activation of the ISR in response to leucine deprivation results in the induction of *p19Arf*.

### Loss of *p19Arf* promotes fibroblast survival during leucine deprivation

**Fig. 2.**
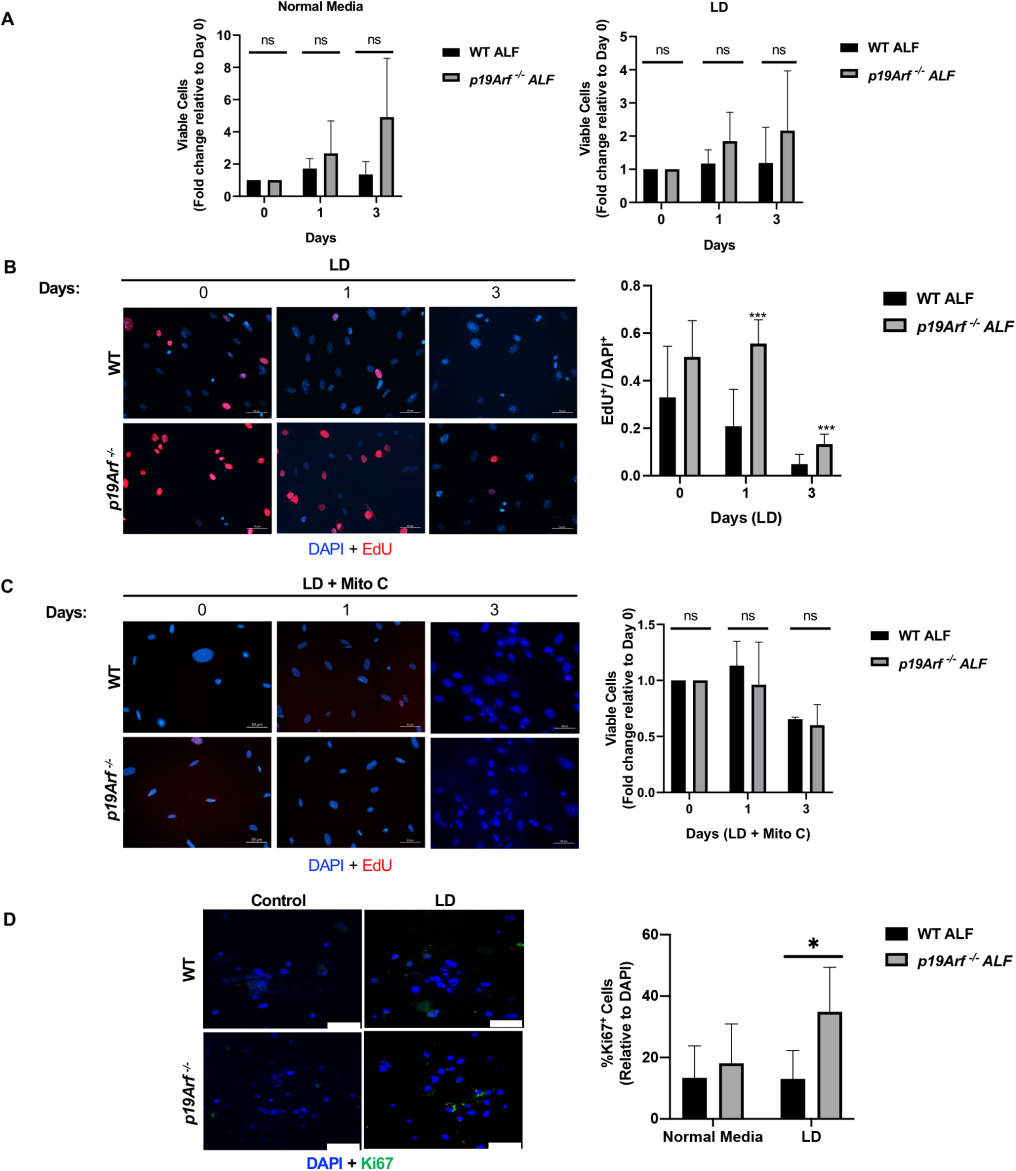
**Loss of *p19Arf* enhances fibroblast proliferation and tumor cell proliferation during leucine deprivation (LD).** (A) Quantification of trypan blue negative WT or *p19Arf^−/−^* lung fibroblasts on the days indicated after normal media (left) or LD media (right). Fold change is relative to day 0 cell counts. (B) Representative images of EdU uptake (16-h pulse) by WT or *p19Arf^−/−^* lung fibroblasts after LD for the indicated days. Scale bars: 50 µm. (C) Representative images of EdU uptake by WT or *p19Arf^−/−^* lung fibroblasts during LD plus mitomycin C (Mito C). Fold change is relative to day 0. Scale bars: 50 μm. (D) Quantification of Annexin V^+^ WT or *p19Arf^−/−^*fibroblasts in the presence of complete or LD media. *N*=3. (E) Representative images of dissociated cells from tumoroids stained for Ki67 Quantification is shown on the right. Scale bars: 50 µm. *N*=5; *, *P*<0.05; ***, *P*<0.001; ns, not significant.

In response to AAD over days rather than hours, we show a trend of reduced viability of WT ALFs in contrast to *p19Arf^−/−^* ALFs, which continue to proliferate even up to 3 days of leucine deprivation (Fig. 2A). The loss of *p19Arf* in mouse embryonic fibroblasts increases proliferation, due in part to the loss of p53 function and loss of cell cycle checkpoints (Weber et al., 2000; Sherr, 2006). We investigated whether *P19Arf* deletion enhances ALF survival during long-term AAD as a result of increased proliferation by pulsing cells with EdU. Our studies confirm a statistically significant increase in the proliferation rate of *p19Arf^−/−^* ALFs as compared to WT fibroblasts (Fig. 2B). To determine whether increased survival of *p19Arf^−/−^* ALFs was primarily due to increased proliferation, we treated fibroblasts with mitomycin C to inhibit DNA synthesis and exposed ALFs to long-term leucine deprivation, revealing that mitomycin C treated *p19Arf^−/−^* fibroblasts die during long-term leucine deprivation (Fig. 2C). These data suggest that the survival of *p19Arf^−/−^* fibroblasts during long-term leucine deprivation is dependent on their increased rates of proliferation.

*P19Arf* activation can induce either apoptosis or senescence (Stott et al., 1998; Suzuki et al., 2003; Yetil et al., 2015), thus we stained *p19Arf*-null ALFs for Annexin V or senescence associated β-Galactosidase (SA-β-Gal) after AAD to detect apoptosis or senescence, respectively. Our data show similar levels of Annexin V staining between leucine-deprived WT and *p19Arf^−/−^* ALFs, indicating that apoptosis in response to AAD was unaffected by *p19Arf* status (Fig. S1A). Additionally, we found no differences in SA-β-Gal staining between WT or *p19Arf^−/−^* ALFs (Fig. S1B,C). To assess the tumor-promoting capabilities of *p19Arf^−/−^* ALFs, we generated three-dimensional (3D) tumoroids consisting of tumor cells co-cultured with either WT or *p19Arf-*null ALFs to examine the effect of ALFs on tumor growth. WT or *p19Arf^−/−^* ALFs were seeded in basement membrane extract. Lewis lung carcinoma (LLC) cells were cultured in 3D on top of basement-membrane extract containing fibroblasts that were either complete or leucine deprived. After 5 days, tumoroids were dissociated into single-cell suspensions and cell proliferation was assayed by staining for Ki67. There was a significant increase in Ki67 staining in tumoroids co-cultured with *p19Arf^−/−^* ALFs during leucine deprivation as compared to LLCs co-cultured with WT ALFs in leucine-deprived media (Fig. 2D).

### *P19Arf^−/−^* fibroblasts show increased activation during leucine deprivation

**Fig. 3.**
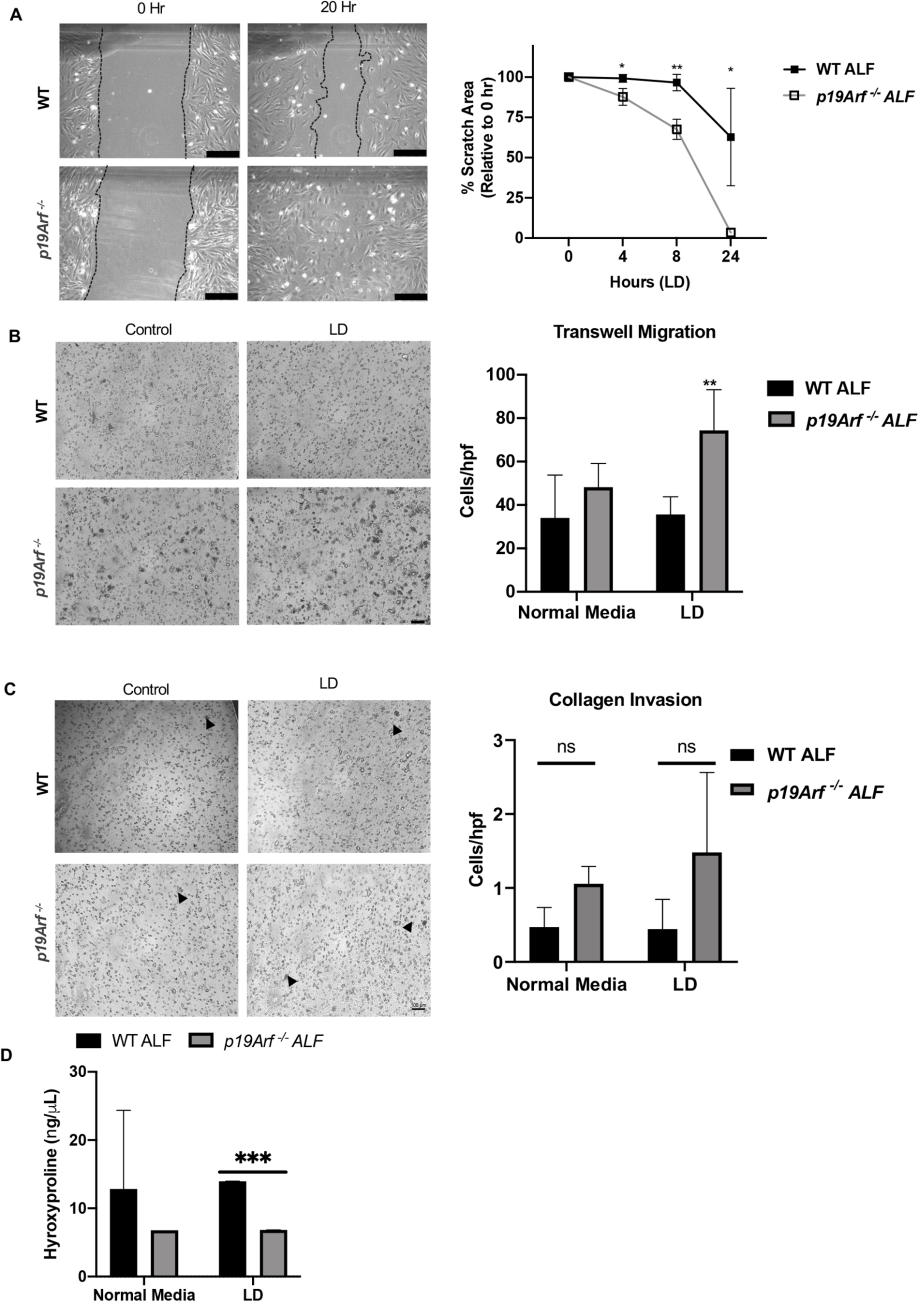
***p19Arf*-null fibroblasts show increased activation during leucine deprivation (LD).** (A) Representative images and quantification of scratch closure by WT or *p19Arf^−/−^* fibroblasts in the presence of complete or LD media. Scratch closure rate was calculated at the indicated times relative to 0 h. *N*=3, *, *P*<0.05. (B) Representative images and quantification of fibroblast transwell migration. WT or *p19Arf^−/−^* fibroblasts were placed in serum-free media with or without LD in the top chamber of transwells and migrated toward complete serum-containing media in lower chamber. Migration was assessed after 24 h by staining with crystal violet. Quantification of migrated cells per high powered field (hpf) is on right. *N*=3, *, *P*<0.05. (C) Representative images and quantification of collagen invasion assays. Fibroblast in serum-free normal or leucine-free media were placed in the top chamber of transwells coated with type 1 rat tail collagen and invaded through the collagen bed toward complete serum-containing media in the lower chamber. Invasion was assessed after 72 h by staining with crystal violet. (D) Quantification of hydroxyproline levels in conditioned media of WT and *p19Arf^−/−^* fibroblasts during collagen invasion assays in serum-free media with or without LD. *N*=3; *, *P*<0.05; **, *P*<0.01; ***, *P*<0.001; ns, not significant; scale bars: 100 μM.

Fibroblast activation in response to injury can be modeled in a scratch assay with ALFs seeded in tissue culture dishes to confluency and then denuded with a pipet tip. We show that during leucine deprivation, *p19Arf^−/−^* fibroblasts cover the denuded area significantly faster than WT fibroblasts indicating increased migration in the presence of leucine deprived media (Fig. 3A). Similarly, *p19Arf^−/−^* ALFs migrated significantly more rapidly through a transwell in response to serum during leucine deprivation further indicating that loss of *p19Arf* enhances fibroblast activation (Fig. 3B). Invasion assays with ALFs traveling through a collagen bed revealed a trend of increased invasion by *p19Arf^−/−^* ALFs as compared to WT ALFs that was maintained during leucine deprivation suggesting an increase in collagen degradation (Fig. 3C). In collagen remodeling, ALFs both degrade and deposit collagen. Since hydroxyproline is a key component of collagen synthesis, hydroxyproline levels were measured in the conditioned media during invasion assays revealing a statistically significant reduction in hydroxyproline in conditioned media from *p19Arf^−/−^* ALFs during leucine deprivation (Fig. 3D). Since collagen synthesis was measured during invasion assays, one interpretation may be that during invasion assays through a collagen bed, collagen synthesis is decreased as fibroblasts are degrading the collagen matrix. Our data indicate that *p19Arf^−/−^* ALFs show increased activation through enhanced migration, invasion through collagen, and decreased collagen synthesis as compared to WT ALFs in response to AAD. These results suggest that loss of *p19Arf* increases fibroblast activation during leucine deprivation.

### Loss of *p19Arf* increases autophagic flux in fibroblasts during leucine deprivation

**Fig. 4.**
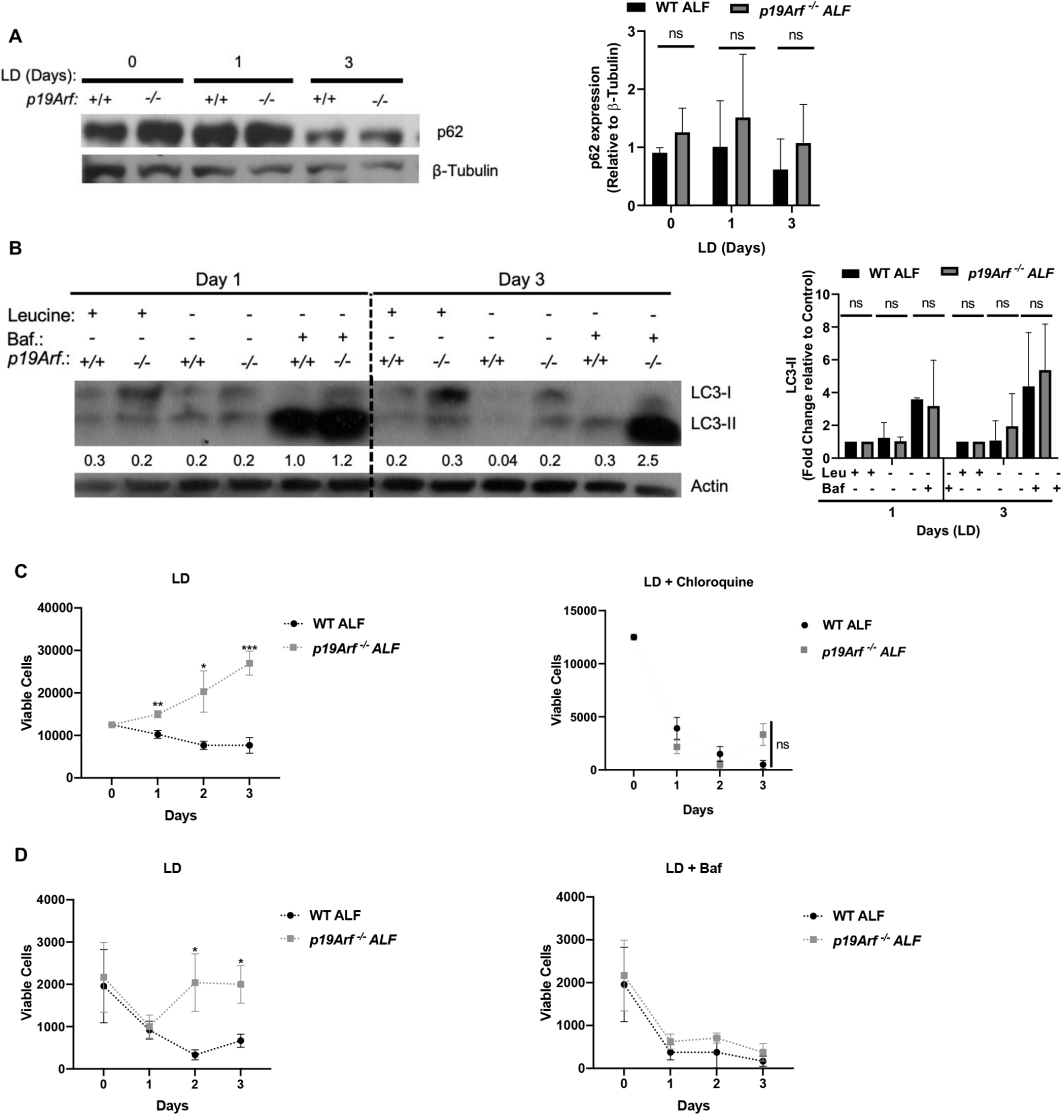
**Loss of *p19Arf* in fibroblasts increases autophagy during leucine deprivation (LD).** (A,B) Western blot analysis of autophagy markers (A) p62 and (B) LC3-I to LC-3II conversion in WT and *p19Arf*^−/−^ fibroblasts during LD for the indicated days. Cells were treated with bafilomycin (baf) or vehicle for 3 h before harvesting. (C,D) Fibroblast cell numbers in the presence of the autophagy inhibitors (C) chloroquine [100 nM] or (D) bafilomycin [1 nM] during LD for the indicated days. *N*=3; *, *P*<0.05; **, *P*<0.01; ***, *P*<0.001; not significant.

Previous studies have shown that cells induce autophagy to recycle organelles and obtain nutrients required for survival during AAD. To investigate whether survival of *p19Arf-*null fibroblasts during leucine deprivation rely on autophagy, we examined expression of the SQSTM1/p62 cargo receptor protein, and the dynamic processing of microtubule-associated proteins 1A/1B light chain 3B (LC3) from LC3-I to LC3-II by western blot to assay autophagic flux. We show a trend of increased p62 expression in *p19Arf-*null ALFs during prolonged AAD as compared to WT ALFs (Fig. 4A). Similarly, we find an increase in LC3-II levels in *p19Arf-null* ALFs at both baseline and in response to AAD (Fig. 4B). Autophagy can be pharmacologically inhibited and the accumulation of autophagy machinery that is processed can be quantified. Bafilomycin inhibits autophagy by raising the pH of the autophagolysosome to prevent degradation of its contents resulting in the accumulation of autophagic markers in a time-dependent manner. Our data show a trend of increased LC3-II expression with bafilomycin treatment after 3 days of leucine deprivation in *p19Arf-null* fibroblasts (Fig. 4B). Fibroblasts were subjected to leucine deprivation and treated with two different autophagy inhibitors, bafilomycin or chloroquine, to assess the contribution of autophagy on ALF survival. The survival benefit of *p19Arf^−/−^* ALFs during leucine deprivation is lost when autophagy is inhibited with either chloroquine (Fig. 4C) or bafilomycin (Fig. 4D) confirming that *p19Arf*^−/−^ ALF are dependent on autophagy to promote their survival through enhanced autophagic flux.

## DISCUSSION

In this study, we investigated the role of the tumor suppressor p19^Arf^ in adult lung fibroblasts in response to stress. While *19Arf* is induced in response to cellular stresses such as oncogenic activation, hypoxia and DNA damage, its response to nutrient deprivation has not been investigated. Our data demonstrate that *p19Arf* is upregulated in fibroblasts in response to leucine deprivation and that loss of *p19Arf* in fibroblasts offers a survival advantage over WT fibroblasts as well as increased migration and activation during prolonged leucine deprivation. Survival of *p19Arf^−/−^* ALFs during long-term leucine deprivation requires autophagy and increased proliferation as pharmacologic inhibition of either autophagy or mitosis prevents survival of *p19Arf*-null ALFs during long-term leucine deprivation.

In response to nutrient deprivation, cells use the process of autophagy to promote survival. We show here that autophagy is increased in *p19Arf-*null ALFs compared to WT ALFs during leucine deprivation as evidenced by the conversion of LC3-I to LC3-II and the degradation of p62. Interestingly, we find that *p19Arf^−/−^* fibroblasts, even at baseline, expressed higher levels of p62 and LC3-II that was even further increased when autophagy was inhibited, suggesting that *p19Arf^−/−^* fibroblasts enhance autophagy to promote their survival. Using two well-characterized autophagy inhibitors bafilomycin and chloroquine, we confirmed the importance of autophagy to *p19Arf-*null fibroblast survival during prolonged leucine deprivation. The decreased survival of *p19Arf-*null ALFs during leucine deprivation upon inhibition of autophagy with either bafilomycin or chloroquine confirms the necessity of autophagy to promote survival under nutrient deprivation conditions. Further studies will be needed to determine how *p19Arf* may directly or indirectly regulate autophagy to inhibit fibroblast survival under leucine deprivation.

A known regulator of autophagy is the mTOR pathway, which primarily regulates cell proliferation and is dependent on the availability of nutrients. In normal conditions, the mTOR pathway, specifically mTOR complex I (mTORC1) regulates mRNA translation, protein turnover, and cellular metabolism. The mTOR pathway is known to be manipulated in cancer, and components of the pathway have been shown to degrade p19^ARF^ to promote proliferation in MEFs (Nakagawa et al., 2015). Further studies will investigate the effect *p19Arf* loss on the mTOR pathway and its regulation on cell processes including proliferation, metabolism, mRNA translation, and protein turnover. As a potent activator of the mTOR pathway and regulator of mTOR directed autophagy (Son et al., 2020), investigating the effects of leucine deprivation on the pathway in the context of *p19Arf* ablation is also an area of interest.

Collectively, our data demonstrate a role for *p19Arf* in fibroblast activation by showing that loss of *p19Arf* promotes fibroblast survival and activation during leucine deprivation, leading to a hyper-activated state in primary adult lung fibroblasts. Further elucidation of the specific downstream targets of *p19Arf* in fibroblasts will provide insight into the link between loss of 19^Arf^ and activation of autophagy during long-term leucine deprivation and may provide new pharmacological targets in the stroma.

## MATERIALS AND METHODS

### Primary lung fibroblast isolation

Lungs from 3- to 5-week-old mice were dissociated in Hank's Balanced Salt Solution containing 5 mg/mL type II collagenase (Worthington, LS004176), 0.5 mg/mL deoxyribonuclease I (Sigma-Aldrich, DN25-1G) and filtered through 100 and 40 µm filters to obtain single-cell suspensions. Fibroblasts were cultured for 1–2 h at 37°C then nonadherent cells were washed off, leaving fibroblasts attached to tissue culture dishes. Fibroblast identity was confirmed by immunostaining for vimentin (Santa Cruz Biotechnology, sc-7557), CD45 (BD Pharmingen, 553771), and CD31 (BD Pharmingen, 553370, 1:100), followed by secondary antibody (Alexa Fluor 647 anti-goat IgG, Alexa Fluor 488 anti-rat IgG, Alexa Fluor 555 streptavidin; Invitrogen, A-21447, A-11006, Thermo Fisher Scientific, 21381, respectively). Fibroblasts were >99% vimentin-positive, <5% CD45 and CD31-positive.

### Cell culture

Fibroblasts were cultured in DMEM-F12+L-glutamine/ penicillin-streptomycin with 10% FBS. LLC cells were purchased from ATCC and sarcoma cells with oncogenic Kras, p53 loss and expression of yellow fluorescent protein (referred to as SKPY) were obtained from Dr. Celeste Simon's lab (University of Pennsylvania, PA, USA) and cultured in DMEM+L-glutamine/penicillin-streptomycin with 10% FBS. Leucine and glutamine-free media (MP Biomedical, 1642149) was supplemented with L-glutamine and 10% FBS.

### Cell cycle arrest

Fibroblasts were treated with Mitomycin C (Sigma-Aldrich, M4287-2MG) at a final concentration of 4 µg/mL in culture media and incubated for 6 h before washing and allowing to recover in complete media. Cell cycle arrest was confirmed through lack of EdU incorporation.

### Transwell migration and invasion

Transwell inserts of 6.5 µm (24 well) with a pore size of 8 µm (Cell Treat, 230639) were used. 5×10^4^ WT or *p19Arf^−/−^* fibroblasts were plated in inserts containing normal or leucine deprived serum-free media in the top chamber and complete 10% serum in the bottom chamber. For migration assays, inserts were uncoated; for invasion assays, inserts were coated with 75 µL 1 mg/mL neutralized type I rat tail collagen for 1 h before cell seeding. For invasion assays, uncoated transwells were used as positive controls; for migration and invasion assays, serum-free media was placed in the bottom chamber as negative control. Migration and invasion transwells were harvested at 3 and 72 h, respectively. The top chamber was then wiped with a cotton swab to remove remaining cells, washed with PBS, fixed, stained with 0.5% crystal violet in 25% methanol solution for 15 min, washed in deionized water, and dried before imaging. Images were taken by tile-scanning using a Zeiss Axio Imager M2 upright microscope with Zen Pro software. Five random images were taken from each transwell, and stained, migrated cells were counted. Assays were performed in triplicate and experiments repeated three times.

### Proliferation assays

A total of 12.5×10^3^ WT or *p19Arf^−/−^* fibroblasts were plated in triplicate in gelatin-coated 12-well plates in complete media for 1–2 h. Day 0 counts were taken, then 10% FBS+DMEM-F12 culture media or 10% FBS+DMEM without leucine was added. On indicated days, triplicate wells were collected, resuspended in equal volume of medium and Trypan blue, and counted on a hemocytometer to assess cell viability.

### Western blot

Cells were lysed in RIPA buffer and equal amounts of protein lysates (10.0–30.0 μg) were separated by 4%–20% gradient polyacrylamide gel (Bio-Rad) electrophoresis then transferred to Immobilon PVDF Membrane (Millipore Sigma). Membranes were blocked in 5% nonfat milk in TBST for 30 min, incubated with primary antibody for 1 h at room temperature [p19^ARF^ (1:500; Novus Biologicals, NB200-174), ATF4 (1:1000; Cell Signaling Technology, 11815, phospho-eIF2α (Ser51) (1:500; Cell Signaling Technology, 3597) total-eIF2α (1:1000; Cell Signaling Technology, 9722), LC3B (1:1000; Cell Signaling Technology, 2775S), and p62 (1:1000; Cell Signaling Technology, 5114)]. Anti-Beta tubulin (1:1000, 2128) was used as loading control. Horseradish-peroxidase-conjugated anti-rabbit (1:2000; Cell Signaling Technology, 7074), anti-mouse (1:2000; Cell Signaling Technology, 7076), or goat anti-rat (1:2000; Cell Signaling Technology, 7077) was used as a secondary antibody. Bands were visualized using enhanced chemiluminescence reagent.

### GCN2 inhibition

Fibroblasts were treated with GCN2-IN-1 (MedChem Express, HY-100877) at a final concentration of 1 µM in leucine deprived media overnight and cells harvested for analysis.

### Autophagy inhibition

Fibroblasts were exposed to leucine deprived media and exposed to Bafilomycin (Cayman Chemicals, 11038) at a final concentration of 1 nM in DMSO 2 h before harvesting lysates and probing for LC3. In survival assays, fibroblasts were treated chloroquine (Sigma-Aldrich, C6628) at a final concentration of 100 nM in leucine deprived media for the times indicated.

### Subcutaneous xenografts

All animal experiments and subcutaneous xenografts were approved by the Institutional Animal Care and Use Committee at the University of Pennsylvania. Six WT and six *p19Arf^−/−^* (three male and three female mice per group) mice between 6 and 8 weeks old (purchased from Jackson Laboratory) had syngeneic murine sarcoma (SKPY; 1.34×10^5^) cells implanted subcutaneously in their flanks. Prior to injection, cells were grown in complete media (DMEM containing 10% FBS). Cells were collected, resuspended in ice-cold, serum-free DMEM for injection. Tumor volumes were assessed and recorded at the indicated timepoints using caliper measurements. The formula, *V*=(*L*)(*W^2^*) (*π*/6), was used to calculate the tumor volume. Tumors were harvested and flash frozen in OCT compound for further analyses.

### Tumoroid assay

Cancer cells in BME were layered onto a bed of BME bed containing fibroblasts and exposed to complete or leucine deprived media. Three-dimensional tumoroids were imaged every 2 days for a week, and numbers and sizes were recorded and measured. Tumoroids were collected and dissociated to acquire single-cell suspension, concentrated via cytospin, and stained for Ki67 (1:100, Novus Biologicals, NB110-89717) Alexa Fluor 488-doneky-anti-sheep IgG (1:500, Novus Biologicals, NBP1-75446 coverslips were mounted face down onto microscope slides using Vectashield anti-fade mounting medium (Vector Laboratories). Fluorescent images were captured with a laser scanning confocal microscope. Confocal imaging was performed on a Leica TCS SP5 and processed using LAS AF software at 63X.

### Migration assay

Cells were grown to confluency in triplicate in 12-well plates. A scratch was generated with a 200-μL tip across each well and pictures taken at the starting timepoint, and at 2–4 h increments post-scratch until complete scratch closure. The percentage of area that was ‘repaired’ was measured using ImageJ software and plotted as the average of the triplicates with standard deviation (SD). Experiments were repeated a minimum of three times.

### qRT-PCR

Total RNA was processed and extracted with TRIzol reagent (Life Technologies, 15596018) and Direct-zol RNA MicroPrep Kit (Zymo Research, R2060). RT reaction was performed using High-Capacity RNA-to-cDNA Kit (Applied Biosystems, 4387406). qRT-PCR was then performed using SYBR Green Master Mix (Bimake, B21202) and a ViiA7 Real-Time PCR Instrument (Applied Biosystems). SYBR probes were used to quantitate expression of *p19Arf* (forward: 5′ AGA GGA TCT TGA GAA GAG GGC C 3′; reverse: 5′ GCA GTT CGA ATC TGC ACC G 3′). Normalization was performed using the housekeeping genes *18S* (forward: 5′ CAATTACAGGGCCTCGAAAG 3′; reverse: 5′AAACGGCTACCACATCCAAG). The mRNA was measured in triplicates with each experiment repeated twice.

### Hydroxyproline assay

Media was collected from transwells during collagen invasions assays 48 h post-plating. Hydroxyproline levels were measured using the colorimetric Hydroxyproline Assay Kit (Sigma-Aldrich, MAK0081KT) per manufacturer's instructions to determine hydroxyproline content as a surrogate for collagen levels.

### Immunostaining

Cells were seeded onto sterile round coverslips (12 mm) on parafilm coat in 10 cm dishes at a density of 12,500 cells per coverslip. Cells were cultured in their respective media at 37°C, 5% CO_2_ for the times indicated. After treatment, EdU proliferation assays were performed with Click-iT EdU Alexa Fluor 594 Imaging Kit (Invitrogen, C10339) according to manufacturer's instructions; fibroblasts were pulsed with 10 mmol/L EdU for 16–18 h before fixation and staining. Coverslips were mounted face down onto microscope slides using Vectashield antifade mounting medium (Vector Laboratories). Images were acquired with laser scanning microscope Zeiss LSM 510 with 63× objective lens (Carl Zeiss AG). All microscopic parameters were kept constant across samples. At least nine different areas were imaged per sample.

### Annexin V flow cytometry

Cells were plated at 100,000 cells per well of a six-well plate and exposed to complete or leucine deprived media. Cells were collected at indicated timepoints, stained for Annexin-V per manufacturer's instructions (BD Pharmingen, 556547), and analyzed on Attune NxT flow cytometer (Thermo Fisher Scientific).

### β-Galactosidase Staining

Cells were plated at 100,000 cells per well of a six-well plate and exposed to complete or leucine deprived media for 3 days. Cells were stained for β-Galactosidase per manufacturer's instructions (Cell Signaling Technology, 9860S), and counted using a Nikon inverted microscope.

### Statistical analysis

Statistical analyses were performed using GraphPad Prism version 8 software, using unpaired Student’s two-tailed *t*-test. Data are presented as mean±SD of at least three independent experiments. Statistical significance was defined as ***, *P*<0.001; **, *P*<0.01; *, *P*<0.05; ns, not significant.

## Supplementary Material

Supplementary information

## References

[BIO058728C1] Arandkar, S., Furth, N., Elisha, Y., Nataraj, N. B., van der Kuip, H., Yarden, Y., Aulitzky, W., Ulitsky, I., Geiger, B. and Oren, M. (2018). Altered p53 functionality in cancer-associated fibroblasts contributes to their cancer-supporting features. *Proc. Natl. Acad. Sci. U.S.A.*, 115, 6410-6415. 10.1073/pnas.1719076115 29866855 PMC6016816

[BIO058728C2] Asha, K. and Sharma-Walia, N. (2018). Virus and tumor microenvironment induced ER stress and unfolded protein response: From complexity to therapeutics. *Oncotarget* 9, 31920-31936. 10.18632/oncotarget.25886 30159133 PMC6112759

[BIO058728C3] B'Chir, W., Maurin, A.-C., Carraro, V., Averous, J., Jousse, C., Muranishi, Y., Parry, L., Stepien, G., Fafournoux, P. and Bruhat, A. (2013). The eIF2α/ATF4 pathway is essential for stress-induced autophagy gene expression. *Nucleic Acids Res.* 41, 7683-7699. 10.1093/nar/gkt563 23804767 PMC3763548

[BIO058728C4] Battu, S., Minhas, G., Mishra, A. and Khan, N. (2017). Amino acid sensing via general control nonderepressible-2 kinase and immunological programming. *Front. Immunol.* 8, 1-11. 10.3389/fimmu.2017.01719 29321774 PMC5732134

[BIO058728C5] Beacham, D. A. and Cukierman, E. (2005). Stromagenesis: The changing face of fibroblastic microenvironments during tumor progression. *Semin. Cancer Biol.* 15, 329-341. 10.1016/j.semcancer.2005.05.003 15970443

[BIO058728C6] Cleveland, J. L. and Sherr, C. J. (2004). Antagonism of Myc functions by Arf. *Cancer Cell* 6, 309-311. 10.1016/j.ccr.2004.09.020 15488753

[BIO058728C7] Fiori, M. E., Di Franco, S., Villanova, L., Bianca, P., Stassi, G. and De Maria, R. (2019). Cancer-associated fibroblasts as abettors of tumor progression at the crossroads of EMT and therapy resistance. *Mol. Cancer* 18, 70. 10.1186/s12943-019-0994-2 30927908 PMC6441236

[BIO058728C8] Gwangwa, M. V., Joubert, A. M. and Visagie, M. H. (2018). Crosstalk between the Warburg effect, redox regulation and autophagy induction in tumourigenesis. *Cell. Mol. Biol. Lett.* 23, 20. 10.1186/s11658-018-0088-y 29760743 PMC5935986

[BIO058728C9] Hanahan, D. and Weinberg, R. A. (2011). Hallmarks of cancer: the next generation. *Cell* 144, 646-674. 10.1016/j.cell.2011.02.013 21376230

[BIO058728C10] Horiguchi, M., Koyanagi, S., Okamoto, A., Suzuki, S. O., Matsunaga, N. and Ohdo, S. (2012). Stress-regulated transcription factor ATF4 promotes neoplastic transformation by suppressing expression of the INK4a/ARF cell senescence factors. *Cancer Res.* 72, 395-401. 10.1158/0008-5472.CAN-11-1891 22102693

[BIO058728C11] Itahana, K. and Zhang, Y. (2008). Mitochondrial p32 is a critical mediator of ARF-induced apoptosis. *Cancer Cell* 13, 542-553. 10.1016/j.ccr.2008.04.002 18538737 PMC4504427

[BIO058728C12] Julia, T., Zhang, P. J., Bi, Y., Satija, C., Marjumdar, R., Stephen, T. L., Lo, A., Chen, H., Mies, C., June, C. H. et al. (2013). Fibroblast activation protein expression by stromal cells and tumor-associated macrophages in human breast cancer. *Hum. Pathol.* 44, 2549-2557. 10.1016/j.humpath.2013.06.016 24074532 PMC4283499

[BIO058728C13] Kalluri, R. (2016). The biology and function of fibroblasts in cancer. *Nature Reviews Cancer* 16, 582-598. 10.1038/nrc.2016.73 27550820

[BIO058728C14] Karimian, A., Ahmadi, Y. and Yousefi, B. (2016). Multiple functions of p21 in cell cycle, apoptosis and transcriptional regulation after DNA damage. *DNA Repair* 42, 63-71. 10.1016/j.dnarep.2016.04.008 27156098

[BIO058728C15] Lehman, S. L., Ryeom, S. and Koumenis, C. (2015). Signaling through alternative Integrated Stress Response pathways compensates for GCN2 loss in a mouse model of soft tissue sarcoma. *Sci. Rep.* 5, 1-13. 10.1038/srep11781. PMC4485314 26123823

[BIO058728C16] Linares, J. F., Cordes, T., Duran, A., Reina-Campos, M., Valencia, T., Ahn, C. S., Castilla, E. A., Moscat, J., Metallo, C. M. and Diaz-Meco, M. T. (2017). ATF4-induced metabolic reprograming is a synthetic vulnerability of the p62-deficient tumor stroma. *Cell Metab.* 26, 817-829.e6. 10.1016/j.cmet.2017.09.001 28988820 PMC5718961

[BIO058728C43] Ishii, N., Maier, D., Merlo, A., Tada, M., Sawamura, Y., Diserenes, A. C. and Van Meir, E. G. (1999). Frequent co-alterations of TP53, p16/CDKN2A, p14ARF, PTEN tumor suppressor genes in human glioma cell lines. *Brain Pathol*. 9, 4690479. 10.1111/j.1750-3639.1999.tb00536.x PMC8098486 10416987

[BIO058728C19] Mejlvang, J., Olsvik, H., Svenning, S., Bruun, J.-A., Abudu, Y. P., Larsen, K. B., Brech, A., Hansen, T. E., Brenne, H., Hansen, T. et al. (2018). Starvation induces rapid degradation of selective autophagy receptors by endosomal microautophagy. *J. Cell Biol.* 217, 3640-3655. 10.1083/jcb.201711002 30018090 PMC6168274

[BIO058728C20] Nakagawa, T., Araki, T., Nakagawa, M., Hirao, A., Unno, M. and Nakayama, K. (2015). S6 kinase- and β-TrCP2-dependent degradation of p19 Arf Is required for cell proliferation. *Mol. Cell. Biol.* 35, 3517-3527. 10.1128/MCB.00343-15 26240281 PMC4573705

[BIO058728C21] New, J., Arnold, L., Ananth, M., Alvi, S., Thornton, M., Werner, L., Tawfik, O., Dai, H., Shnayder, Y., Kakarala, K. et al. (2018). Secretory Autophagy in cancer-associated fibroblasts promotes head and neck cancer progression and offers a novel therapeutic target. 77, 6679-6691. 10.1158/0008-5472.CAN-17-1077.Secretory 10.1158/0008-5472.CAN-17-1077 PMC5712244 28972076

[BIO058728C22] Ozenne, P., Eymin, B., Brambilla, E. and Gazzeri, S. (2010). The ARF tumor suppressor: structure, functions and status in cancer. *Int. J. Cancer* 127, 2239-2247. 10.1002/ijc.25511 20549699

[BIO058728C23] Pakos-Zebrucka, K., Koryga, I., Mnich, K., Ljujic, M., Samali, A. and Gorman, A. M. (2016). The integrated stress response. *EMBO Rep.* 17, 1374-1395. 10.15252/embr.201642195 27629041 PMC5048378

[BIO058728C25] Qiu, W., Hu, M., Sridhar, A., Opeskin, K., Fox, S., Shipitsin, M., Trivett, M., Thompson, E. R., Ramakrishna, M., Gorringe, K. L. et al. (2008). No evidence of clonal somatic genetic alterations in cancer-associated fibroblasts from human breast and ovarian carcinomas. *Nat. Genet.* 40, 650-655. 10.1038/ng.117 18408720 PMC3745022

[BIO058728C26] Santos, A. M., Jung, J., Aziz, N., Kissil, J. L. and Puré, E. (2009). Targeting fibroblast activation protein inhibits tumor stromagenesis and growth in mice. *J. Clin. Investig.* 119, 3613-3625. 10.1172/JCI38988 19920354 PMC2786791

[BIO058728C27] Sharpless, N. E. and Sherr, C. J. (2015). Forging a signature of in vivo senescence. *Nature Reviews Cancer* 15, 397-408. 10.1038/nrc3960 26105537

[BIO058728C28] Sheen, J.-H., Zoncu, R., Kim, D. and Sabatini, D. M. (2011). Defective regulation of autophagy upon leucine deprivation reveals a targetable liability of human melanoma cells in vitro and in vivo. *Cancer Cell* 19, 613-628. 10.1016/j.ccr.2011.03.012 21575862 PMC3115736

[BIO058728C29] Sherr, C. J. (2006). Divorcing ARF and p53: an unsettled case. *Nature Reviews Cancer* 6, 663-673. 10.1038/nrc1954 16915296

[BIO058728C30] Skardal, A., Mack, D., Atala, A. and Soker, S. (2013). Substrate elasticity controls cell proliferation, surface marker expression and motile phenotype in amniotic fluid-derived stem cells. *Journal of the Mechanical Behavior of Biomedical Materials* 17, 307-316. 10.1016/j.jmbbm.2012.10.001 23122714 PMC3665276

[BIO058728C31] Son, S. M., Park, S. J., Stamatakou, E., Vicinanza, M., Menzies, F. M. and Rubinsztein, D. C. (2020). Leucine regulates autophagy via acetylation of the mTORC1 component raptor. *Nat. Commun.* 11, 1-13. 10.1038/s41467-020-16886-2. 32561715 PMC7305105

[BIO058728C32] Stott, F. J., Bates, S., James, M. C., McConnell, B. B., Starborg, M., Brookes, S., Palmero, I., Ryan, K., Hara, E., Vousden, K. H. et al. (1998). The alternative product from the human CDKN2A locus, p14(ARF), participates in a regulatory feedback loop with p53 and MDM2. *EMBO J.* 17, 5001-5014. 10.1093/emboj/17.17.5001 9724636 PMC1170828

[BIO058728C33] Suzuki, H., Kurita, M., Mizumoto, K., Nishimoto, I., Ogata, E. and Matsuoka, M. (2003). p19ARF-induced p53-independent apoptosis largely occurs through BAX. *Biochem. Biophys. Res. Commun.* 312, 1273-1277. 10.1016/j.bbrc.2003.11.071 14652011

[BIO058728C34] Tang, X., Keenan, M. M., Wu, J., Lin, C.-A., Dubois, L., Thompson, J. W., Freedland, S. J., Murphy, S. K., Chi, J.-T. and Curran, S. P. (2015). Comprehensive profiling of amino acid response uncovers unique methionine-deprived response dependent on intact creatine biosynthesis. *PLoS Genet.* 11, e1005158. 10.1371/journal.pgen.1005158 25849282 PMC4388453

[BIO058728C35] Wang, L., Cao, L., Wang, H., Liu, B., Zhang, Q., Meng, Z., Wu, X., Zhou, Q. and Xu, K. (2017). Cancer-associated fibroblasts enhance metastatic potential of lung cancer cells through IL-6/STAT3 signaling pathway. *Oncotarget* 8, 76116-76128. 10.18632/oncotarget.18814 29100297 PMC5652691

[BIO058728C36] Weber, J. D., Jeffers, J. R., Rehg, J. E., Randle, D. H., Lozano, G., Roussel, M. F., Sherr, C. J. and Zambetti, G. P. (2000). p53-independent functions of the p19 ARF tumor suppressor. *Genes Dev*. 14, 2358-2365. 10.1101/gad.827300 10995391 PMC316930

[BIO058728C37] White, E. and DiPaola, R. S. (2009). The double-edged sword of autophagy modulation in cancer. *Clin. Cancer Res.* 15, 5308-5316. 10.1158/1078-0432.CCR-07-5023 19706824 PMC2737083

[BIO058728C38] Xiao, F., Wang, C., Yin, H., Yu, J., Chen, S., Fang, J. and Guo, F. (2016). Leucine deprivation inhibits proliferation and induces apoptosis of human breast cancer cells via fatty acid synthase. *Oncotarget* 7, 63679-63689. 10.18632/oncotarget.11626 27579768 PMC5325395

[BIO058728C39] Ye, J., Kumanova, M., Hart, L. S., Sloane, K., Zhang, H., De Panis, D. N., Bobrovnikova-Marjon, E., Diehl, J. A., Ron, D. and Koumenis, C. (2010). The GCN2-ATF4 pathway is critical for tumour cell survival and proliferation in response to nutrient deprivation. *EMBO J.* 29, 2082-2096. 10.1038/emboj.2010.81 20473272 PMC2892366

[BIO058728C40] Yetil, A., Anchang, B., Gouw, A. M., Adam, S. J., Zabuawala, T., Parameswaran, R., van Riggelen, J., Plevritis, S. and Felsher, D. W. (2015). p19ARF is a critical mediator of both cellular senescence and an innate immune response associated with MYC inactivation in mouse model of acute leukemia. *Oncotarget* 6, 3563-3577. 10.18632/oncotarget.2969 25784651 PMC4414137

[BIO058728C41] Ying, S. and Khaperskyy, D. A. (2020). UV damage induces G3BP1-dependent stress granule formation that is not driven by translation arrest via mTOR inhibition. *J. Cell Sci.* 133, jcs.248310. 10.1242/jcs.248310 PMC7648617 32989041

[BIO058728C42] Zindy, F., Williams, R. T., Baudino, T. A., Rehg, J. E., Skapek, S. X., Cleveland, J. L., Roussel, M. F. and Sherr, C. J. (2003). Arf tumor suppressor promoter monitors latent oncogenic signals in vivo. *Proc. Natl. Acad. Sci. U.S.A.*, 100, 15930-15935. 10.1073/pnas.2536808100 14665695 PMC307670

