# Supplementary material for: Loss of p19Arf promotes fibroblast survival during leucine deprivation
Source: Biol Open. 2022 Feb 17;11(2):bio058728. doi: 10.1242/bio.058728 (PMC8864297; doi:10.1242/bio.058728)
Supplement: Supplementary information [file biolopen-11-058728-s1.pdf]

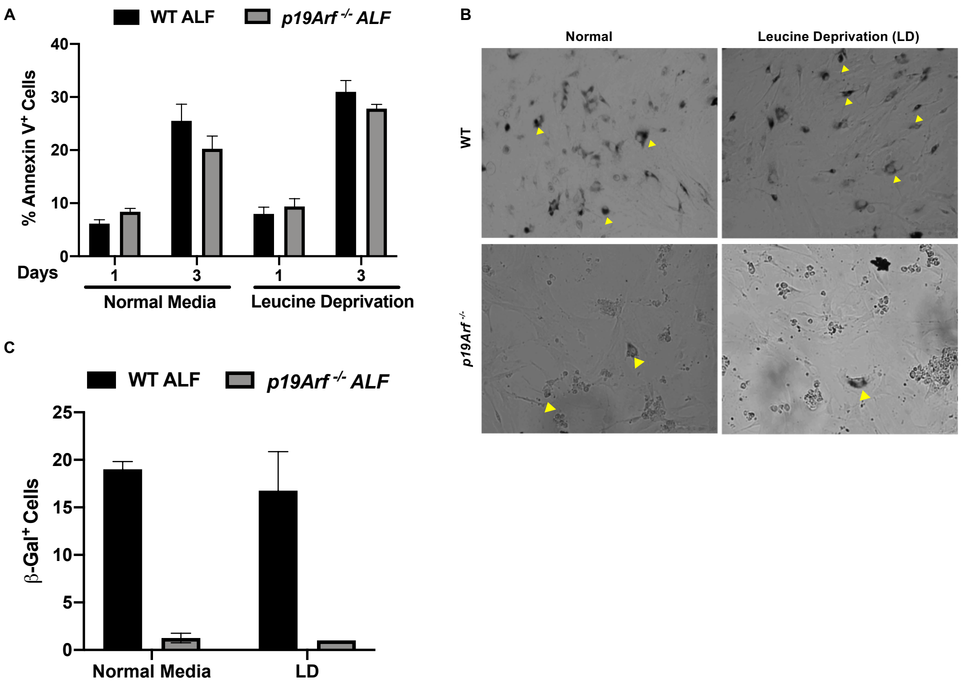

Fig. S1. Loss of *p19Arf* has no effect on apoptosis or senescence induction during leucine deprivation. A. Quantification of Annexin V+ WT or *p19Arf-/-* fibroblasts in the presence of complete or LD media. N=3. **B.** Representative images of WT or *p19Arf-/-* fibroblasts stained for β-Galactosidase (β-Gal) following 3 days in complete or LD media. Yellow arrows = senescent cells. **C.** Quantification of β-Gal + fibroblasts N=3.

Biology Open • Supplementary information
